# Validating cancer-specific and generic quality of life (QoL) instruments among patients with head and neck cancers: incorporating the European Organization for Research and Treatment of Cancer Quality of Life Questionnaire-Head and Neck Module with generic QoL instruments

**DOI:** 10.1186/s41155-026-00394-1

**Published:** 2026-05-06

**Authors:** Mohsen Saffari, Tzu-Yen Chang, Musheer A. Aljaberi, Kah Ying Yap, An-Chi Li, Jung-Sheng Chen, Shi-Qing Wang, Mark D. Griffiths, Chung-Ying Lin

**Affiliations:** 1 Health Research Center, Life Style Institute, Baqiyatallah University of Medical Sciences, Tehran, Iran; 2 Health Education Department, School of Health, Baqiyatallah University of Medical Sciences, Tehran, Iran; 3 Division of Plastic and Reconstructive Surgery, Department of Surgery, College of Medicine, National Cheng Kung University Hospital, National Cheng Kung University, Tainan, Taiwan; 4 Department of Public Health, College of Medicine, National Cheng Kung University, Tainan, Taiwan; 5 Franciscus Academy, Franciscus, Rotterdam, The Netherlands; 6 Institute for Healthcare (IVG), Rotterdam University of Applied Sciences, Rotterdam, The Netherlands; 7 Department of Internal Medicine, Section Nursing Science, Erasmus University Medical Center (Erasmus MC), Rotterdam, The Netherlands; 8 Institute of Allied Health Sciences, College of Medicine, National Cheng Kung University, 1, University Rd, Tainan, 701401 Taiwan; 9 Department of Otolaryngology, E-Da Hospital, I-Shou University, No. 1, Yida Rd., Yanchao Dist, Kaohsiung, Kaohsiung 824005 Taiwan; 10 Department of Medical Research, E-Da Hospital, I-Shou University, Kaohsiung, 824005 Taiwan; 11 Psychology Department, Nottingham Trent University, Nottingham, UK; 12 Biostatistics Consulting Center, National Cheng Kung University Hospital, College of Medicine, National Cheng Kung University, Tainan, 701401 Taiwan; 13 Department of Occupational Therapy, College of Medicine, National Cheng Kung University, Tainan, 701401 Taiwan; 14 School of Nursing, College of Nursing, Kaohsiung Medical University, Kaohsiung, Taiwan

**Keywords:** Cancer, Confirmatory factor analysis, EQ-5D, Psychometrics, WHOQOL-BREF

## Abstract

**Purpose:**

Head and neck cancers can significantly affect health-related quality of life (QoL). Using a novel approach, the present study validated a Taiwanese version of the revised European Organization for Research and Treatment of Cancer Quality of Life Questionnaire-Head and Neck Module (QLQ-HN43).

**Methods:**

The present study was a cross-sectional study with the data collected from November 2021 to March 2022, and included 290 participants (82.7% males, mean age = 58.9 years [SD = 11.03]) from a medical center. Internal consistency was examined using Cronbach’s α. Confirmatory factor analysis (CFA) was conducted testing different models. Two generic QoL measures (the World Health Organization Quality of Life Questionnaire Brief version [WHOQOL-BREF] and EuroQol Five-Dimension [EQ-5D]), were used to assess concurrent validity. Known-groups validity was used to indicate how the QLQ-HN43 could differentiate individuals with mild-to-moderate problems from those with severe problems associated with QoL.

**Results:**

Internal consistency was acceptable for QLQ-HN43 subscales (.68 < α < .94). CFA results showed appropriate fit indices, particularly for each individual model and the incorporated model containing both QLQ-HN43 and WHOQOL-BREF. The concurrent validity of the QLQ-HN43 was supported by the significant correlations with WHOQOL-BREF (*r*=-.111 to − .508; *p*-values<.05) and EQ-5D utility in CFA findings (*r*=.276 to .655; *p*-values<.01). Significant differences were found between those at the initial stages of the disease and those at the advanced stage, supporting known-group validity.

**Conclusion:**

The QLQ-HN43 scale has acceptable psychometric properties for Taiwanese individuals with head or neck cancers. The feasibility of incorporating EQ-5D with the QLQ-HN43 needs further investigation.

**Supplementary Information:**

The online version contains supplementary material available at 10.1186/s41155-026-00394-1.

## Introduction

Head and neck cancers (HNCs) affect a considerable number of individuals worldwide, with nearly one million new cases diagnosed annually, and approximately one-third resulting in death (Gormley et al., 2022; Sun et al., 2025). HNCs are among the ten most common malignancies globally and can adversely impact essential body functions such as eating, breathing, and speaking (Heinolainen et al., 2025). They can often lead to complications such as dysphagia and dysphonia, and may also affect surrounding structures, including salivary and thyroid glands, oral and nasal cavities, skull, face, ears, and eyes (Fillo et al., 2026; Parsons & Dewan, 2025; Taylor et al., 2023). Consequently, HNCs can have profound negative effects on the general health status of those affected (Fillo et al., 2026; Wu et al., 2025, 2026).

Although treatment options have advanced based on disease stage, routine curative procedures such as surgery and radiotherapy that would be effective, particularly in early stages, may include negative impacts on patients’ physical, emotional, and social aspects of well-being (El Abiddine et al., 2025; Sabah et al., 2025; Sharma et al., 2024). These treatments may lead to long-lasting reductions in health-related quality of life (QoL), even after treatment (Fillo et al., 2026; Caetano et al., 2022; So et al., 2012).

QoL is a multidimensional construct reflecting individuals’ perceptions of their physical, psychological, and social well-being. The World Health Organization defines QoL as individuals’ perceptions of their position in life within the context of their culture and value systems, and in relation to their goals, expectations, standards, and concerns. This definition remains widely adopted in contemporary research, where QoL is recognized as a subjective and comprehensive indicator of overall well-being beyond traditional health measures (Liem et al., 2025; The WHOQOL Group, 1998). In terms of health-associated aspects, QoL provides a multidimensional indicator of health that explains how illness and treatment influence personal well-being and physical function (Suasnabar et al., 2024). This measure goes beyond traditional health metrics such as morbidity and mortality, offering insights into various physical, mental, and social dimensions of life (Nolazco & Chang, 2023). QoL has been recognized as a primary outcome for evaluating the curative effects of interventions or treatments among patients with different types of cancer, including HNCs (Deana et al., 2025; Nolazco & Chang, 2023; Wu et al., 2024).

Given that HNCs substantially affect an individual’s health status, and that these conditions may affect quality of life, there is a growing need to use valid and reliable instruments to assess health-related outcomes such as QoL, particularly for those undergoing treatment (Adamowicz et al., 2022; Koffi et al., 2025; Tonsbeek et al., 2024; Grusdat et al., 2022). HNCs differ from other malignancies due to anatomical and physiological changes that typically occur in advanced stages, leading to distinct symptoms and associated complications, as well as psychological problems (Adamowicz et al., 2022; Koffi et al., 2025; Taylor et al., 2023; Tonsbeek et al., 2024). These cancers can cause a significant decrease in QoL compared with other malignancies (Adamowicz et al., 2022; Fillo et al., 2026; Koffi et al., 2025; So et al., 2012). In addition, issues such as body image concerns, social isolation, insomnia, and depression are particularly pronounced among HNC patients and contribute to further deterioration of QoL (McDowell et al., 2022; Tseng et al., 2022). Therefore, as highlighted by Adamowicz et al. (2022), Fillo et al. (2026), and So et al. (2012), regular monitoring for physical, psychological, and social components of health, typically included in QoL assessments, is essential for patients undergoing treatment and survivors of HNCs (Adamowicz et al., 2022; Fillo et al., 2026; So et al., 2012).

Several general and specific instruments have been developed to assess QoL among cancer patients (Deana et al., 2025). Although general instruments (i.e., generic QoL instruments) provide a broad basis for assessing QoL across different cancer types, specific instruments are more effective in capturing particular issues and concerns not addressed by generic instruments (Zimmermann et al., 2021). Therefore, applying both generic and specific QoL instruments allows for a comprehensive assessment of QoL among individuals with HNCs, facilitating better healthcare planning and the design of effective health promotion interventions.

In the early 1990s, the European Organization for Research and Treatment of Cancer (EORTC) developed a specific 35-item instrument to assess QoL in HNC patients, initially known as EORTC Quality of Life Questionnaire-Head and Neck Module (Tolstrup et al., 2023). A systematic review in 2022 confirmed its validation across 21 languages with acceptable psychometric properties (Parkar & Sharma, 2022). Following extensive use, the instrument has been revised to the current 43-item version (QLQ-HN43), which serves as a supplementary module to the General Quality of Life Questionnaire Core 30 (QLQ-C30), because the 43-item version also addresses specific issues related to HNCs (e.g., pain and dryness in the mouth, problems with swallowing and teeth, body image concerns, speech, feelings, sexual function, skincare, and fear of disease progression) (Ramalingam et al., 2023). It also includes items related to coughing, mouth opening, social relationships, weight loss, wounds, and psychological consequences (e.g., anxiety) (Singer et al., 2019). Although the EORTC QLQ-HN43 was initially developed with individuals from 18 different countries, it has only been validated linguistically and culturally in a few languages, including English, Serbian, and Marathi (a variant of the language spoken in some areas of India), using adequate samples (Singer et al., 2019; Trivic et al., 2016; Waghmare et al., 2020).

While the EQRTC QLQ-C30 is a generally recommended instrument for assessing QoL among cancer patients, several standard measures can be used alongside the EORTC QLQ-HN43 to provide a more comprehensive understanding of QoL. Previous studies have used scales such as the World Health Organization Quality of Life Questionnaire Brief version (WHOQOL-BREF) and EuroQol Five-Dimension (EQ-5D) as general instruments with promising results, often combined with specific instruments, such as the University of Washington Quality of Life Questionnaire, to capture all dimensions of QoL (Deana et al., 2025; Davies et al., 2020; Ganesan et al., 2019). Using brief and standardized instruments may enhance participant engagement by reducing time and energy demands on patients. Indeed, for patients at advanced stages of cancer or experiencing complications, specific QoL instruments are vital for understanding various aspects of overall QoL (Davies et al., 2020).

To the best of the present authors’ knowledge, no previous studies have validated the QLQ-HN43 as a specific measure, alongside other generic instruments, such as WHOQOL-BREF and EQ-5D, among Taiwanese individuals with HNCs. Therefore, the present study aimed to (i) assess psychometric properties of the QLQ-HN43 (including internal consistency, dimensionality, concurrent validity, and known-group validity), and (ii) examine whether the QLQ-HN43 can be incorporated with generic QoL instruments to assess different aspects of QoL among individuals in this population.

## Methods

### Study design, participants, and procedures

The present study was a cross-sectional study, and its protocol was approved by the Institutional Review Board of the National Cheng Kung University Hospital (approval number: B-ER-109-506) before data collection. The study enrolled all head and neck cancer patients at the medical center in Tainan, Taiwan (i.e., the primary medical center in Southern Taiwan) at the time of the study. Target participants were recruited from November 8, 2021, to March 9, 2022. The inclusion criteria of the participants were (i) being aged 20 years or above, and (ii) having a diagnosis of head and neck cancer within the data collection period, irrespective of whether they were just about to begin their treatment or whether they were survivors who had completed their treatment. Participants were excluded if they could not (i) understand spoken or written Chinese comprehensively, and (ii) provide written informed consent to indicate their willingness to participate.

Several head and neck cancer surgeons first identified eligible patients and introduced the study to their patients. When an eligible patient agreed to participate and provided their informed consent, they were invited to complete three instruments (i.e., EQ-5D, WHOQOL-BREF, and QLQ-HN43; please see the *Instruments* section below for details) in the outpatient clinic. The participants’ demographics and clinical characteristics were then retrieved from the Cancer Registration Database in the medical center and integrated with their self-report data for data analysis.

### Instruments

#### Revised European Organization for Research and Treatment of Cancer Quality of Life Questionnaire-Head and Neck module (QLQ-HN43)

The QLQ-HN43 is a head and neck cancer-specific QoL instrument that assesses multiple QoL domainswithin a psychometric framework (Singer et al., 2019). The QLQ-HN43 is an updated version assessing head and neck cancer-specific QoL (i.e., Module QLQ-H&N35) (Bjordal et al. 2000). The revised QLQ-HN43 contains 43 items across 19 factors. For the following factors, each was assessed using one item: problems with mouth opening, coughing, social contact problems, swelling in the neck, weight loss, problems with wound healing, and neurological problems. For the following factors, each was assessed using two items: dry mouth and sticky saliva, problems with senses, sexual problems, problems with the shoulder, and fear of disease progression.

For the following factors, each was assessed using three items: problems with teeth, body image problems, and skin problems. For the following factors, each was assessed using four items: mouth pain, swallowing problems, and social eating problems. The speech problems factor was assessed using five items (Singer et al., 2019). All the QLQ-HN43 items are rated using a four-point Likert scale from 0 (*never*) to 3 (*very often*). The factor scores are linearly converted from the Likert scale score to a 0-100 scale, where a higher score indicates a lower QoL (Singer et al., 2019). More specifically, item scores within the same domain were first averaged, then divided by 3 and multiplied by 100 to obtain scores on a 0-100 scale. To the best of the present authors’ knowledge, no prior study has specifically validated the QLQ-HN43 among Taiwanese individuals. However, the QLQ-HN43 validation study included participants from different countries, including Taiwan (Singer et al., 2019).

#### World Health Organization Quality of Life Questionnaire Brief version (WHOQOL-BREF)

The WHOQOL-BREF is a generic QoL instrument that assesses four domains of QoL within a psychometric framework (Su et al., 2014). The original version of the WHOQOL-BREF contains 26 items: one item assessing overall QoL, one assessing general health, seven assessing physical QoL, six assessing psychological QoL, three assessing social QoL, and eight assessing environment QoL (The WHOQOL Group, 1998). The WHOQOL-BREF has been translated and culturally adapted into Chinese versions for Taiwanese (i.e., the Taiwan version of WHOQOL-BREF) (Yao et al., 2002). The Taiwanese version of WHOQOL-BREF adds two local cultural items: one in the social domain (respect) and one in the environmental domain (eating) (Yao et al., 2002). All WHOQOL-BREF items are rated using a five-point Likert scale from 1 to 5 (with different descriptors for each item). The domain scores are linearly converted from the Likert scale score to a 4–20 scale, where a higher score indicates a better QoL (Yao et al., 2002). More specifically, the item scores in the same domain are first averaged and then multiplied by 4 to obtain scores on a 4–20 scale. Previous studies have shown that the Taiwanese version of WHOQOL-BREF has satisfactory psychometric properties among cancer patients (Lin et al., 2017, 2019).

#### EuroQol Five-Dimension (EQ-5D)

The EQ-5D is a generic QoL instrument that assesses the entire QoL within a utility framework (Rabin & Charro, 2001). The EQ-5D contains five items assessing mobility, self-care, usual activities, pain/discomfort, and anxiety/depression, and one item assessing overall QoL using a visual analog scale (EUROQOL, 2024). In the present study, each item was rated at one of three levels (i.e., ‘no problems’, ‘some problems’, or ‘extreme problems’) (EUROQOL, 2024). The scores are then converted into a utility score (ranging between 0 and 1; the higher the score, the better the quality of life) according to an algorithm developed using Taiwanese norms (Lee et al., 2013). More specifically, full health is represented using the value 1.000, which is used to deduct a constant term (0.185), and then a given value of a level is deducted for every item. The EQ-5D has been translated and culturally adapted into the Chinese version for Taiwanese individuals (i.e., the Taiwan version of EQ-5D) (Lee et al., 2013). Previous studies have shown that the Taiwanese version of EQ-5D has satisfactory psychometric properties among cancer patients (Lang et al., 2010).

### Data analysis

The participants’ demographics and clinical characteristics were first analyzed using descriptive statistics to understand the features of the participants. The QLQ-HN43 item properties were also analyzed using descriptive statistics. Internal consistency was then evaluated for each subscale of the QLQ-HN43 using Cronbach’s α, where .7 indicates acceptable internal consistency (Chaudhary et al., 2024).

Confirmatory factor analysis (CFA) with a diagonally weighted least squares estimator was used to examine several proposed QoL models, including a model with only cancer-specific QoL (Model 1: using the QLQ-HN43 item scores), two models with both cancer-specific and generic QoL (Model 2: Model 1 plus WHOQOL-BREF domain scores being generic QoL factor; Model 3: Model 1 plus five EQ-5D item scores being generic QoL factor), and four models with one subscale in cancer-specific QoL (Model 4: using four PA items in the QLQ-HN43 as observed variables; Model 5: using four SW items in the QLQ-HN43 as observed variables; Model 6: using five speech problem items in the QLQ-HN43 as observed variables; and Model 7: using four social eating items in the QLQ-HN43 as observed variables). Models 2 and 3 were constructed because they can provide correlations between studied factors in latent scores, and the latent score correlations were additionally used to examine the concurrent validity of the QLQ-HN43. Models 4 to 7 were constructed because all other QLQ-HN43 subscales had only three or fewer items. Because CFA needs to have a minimum of four items (with one underlying factor) for meaningful analysis, only the four subscales that had more than three items were tested. Moreover, specific factor loadings of each QLQ-HN43 item and the latent correlations between QLQ-HN43 subscales were calculated to demonstrate the internal structure of the QLQ-HN43. For all CFA models, the following fit indices with suggested cutoffs were used: a nonsignificant χ^2^, a comparative fit index (CFI) > 0.9, a Tucker-Lewis index (TLI) > 0.9, a root mean square error of approximation (RMSEA) < 0.08, and a standardized root mean square residual (SRMR) < 0.08 (Hunag et al., 2024).

As aforementioned, the concurrent validity of the QLQ-HN43 was evaluated using CFA with the WHOQOL-BREF domains and the EQ-5D utility, using latent scores. In addition, the QLQ-HN43 subscales were specifically evaluated to determine whether they had stronger associations with more relevant WHOQOL-BREF domains than with less relevant WHOQOL-BREF domains. Finally, independent *t*-tests together with Cohen’s *d* (*d* at 0.2 indicates a small effect; 0.5 moderate effect; and 0.8 large effect) (Cohen, 1988) were used to examine the known-group validity of the QLQ-HN43. The participants were classified into two groups according to clinical stage (i.e., stages 0 to III vs. stage IV) to generate larger sample sizes for comparisons. Then, the score differences in each QLQ-HN43 subscale between the two groups were compared. Moreover, the independent *t*-tests together with Cohen’s *d* were applied to compare WHOQOL-BREF domain scores and EQ-5D utility score between the two groups, illustrating how QLQ-HN43 performed differently from WHOQOL-BREF and EQ-5D in known-group validity. All the statistical analyses were conducted using *IBM SPSS 20.0* (IBM Inc., Chicago: IL), except for CFA models which were conducted using the *lavaan* package in *R* software (Rosseel, 2012).

## Results

Of the 290 participants (mean age = 58.91 years; SD = 11.03), over four-fifths were male (*n* = 240; 82.7%). The participants’ detailed demographics and clinical characteristics are shown in Table 1. Regarding the QLQ-HN43 item and scale properties, the range of the raw scores was between 1.28 and 2.15, with all item and subscale scores having acceptable distributions (skewness = 0.44 to 2.36; kurtosis=-0.96 to 6.94). Moreover, all QLQ-HN43 subscales had nearly acceptable to excellent internal consistency (α = .68 to .94), with the entire QLQ-HN43 having excellent internal consistency (α = .96) (Table 2).

**Table 1:** Participants’ characteristics (*N* = 290)

	Mean (SD) or *n* (%)
Demographics	
Age (in years)	58.91 (11.03)
Sex (male)	240 (82.7)
*Educational level*	
* Primary school or below*	43 (14.8)
* Junior high school*	57 (19.7)
* Senior high school*	93 (32.1)
* College or above*	67 (23.1)
* Missing*	30 (10.3)
Marital status (currently married)	196 (67.6)
Religious (no)	30 (10.3)
Monthly household income (> 30,000 NTD)	140 (48.3)
Clinical characteristics	
Clinical stage of cancer	
* 0 or I*	44 (15.2)
* II*	34 (11.7)
* III*	33 (11.4)
* IV*	70 (24.1)
* Missing*	109 (37.6)
Age at cancer diagnosis (in years)	55.36 (10.43)
Duration of cancer (in years)	3.85 (3.58)

*NTD*  New Taiwan Dollar; 30 NTD≈1 USD

**Table 2:** Item and subscale properties using raw scores with scale internal consistency in HN43 (entire HN43 α = .96)

Subscale (item)	Mean (SD)	Loading^a^	Skewness	Kurtosis	Cronbach’s α
PA	1.44 (0.57)	.60	1.64	3.14	.85
(q31)	1.50 (0.72)	.80	1.43	1.71	
(q32)	1.37 (0.62)	.77	1.66	2.34	
(q33)	1.39 (0.62)	.82	1.60	2.56	
(q34)	1.52 (0.76)	.70	1.49	1.80	
SW	1.70 (0.76)	.86	1.28	1.19	.86
(q35)	1.55 (0.84)	.79	1.53	1.54	
(q36)	1.54 (0.83)	.77	1.57	1.79	
(q37)	2.06 (1.09)	.83	0.62	-0.96	
(q38)	1.66 (0.83)	.69	1.22	0.92	
TE	2.01 (0.93)	.73	0.70	-0.50	.86
(q39)	2.03 (1.01)	.72	0.63	-0.70	
(q40)	1.99 (1.04)	.77	0.70	-0.74	
(q73)	2.01 (1.11)	.93	0.69	-0.94	
DR	2.00 (0.81)	.73	0.57	-0.49	.70
(q42)	2.15 (0.96)	.73	0.44	-0.74	
(q43)	1.85 (0.90)	.73	0.81	-0.21	
SE	1.51 (0.72)	.57	1.61	2.46	.68
(q44)	1.44 (0.83)	.57	1.93	2.81	
(q45)	1.58 (0.83)	.90	1.44	1.36	
SP	1.67 (0.81)	.73	1.37	1.10	.93
(q47)	1.72 (0.92)	.73	1.19	0.52	
(q55)	1.61 (0.89)	.92	1.41	1.08	
(q56)	1.61 (0.92)	.85	1.44	1.03	
(q57)	1.72 (0.96)	.85	1.16	0.24	
(q58)	1.68 (0.94)	.87	1.25	0.49	
BI	1.40 (0.64)	.68	1.92	3.91	.92
(q48)	1.41 (0.69)	.91	1.80	2.99	
(q49)	1.41 (0.70)	.90	1.85	3.22	
(q50)	1.40 (0.66)	.86	1.76	3.04	
SO	1.72 (0.86)	.85	1.18	0.52	.93
(q51)	1.88 (1.00)	.91	0.87	-0.41	
(q52)	1.50 (0.87)	.81	1.69	1.83	
(q53)	1.71 (0.95)	.86	1.20	0.38	
(q54)	1.79 (0.98)	.90	1.06	0.02	
SX	1.42 (0.69)	.47	1.89	3.46	.93
(q60)	1.44 (0.73)	.97	1.80	3.01	
(q61)	1.39 (0.70)	.89	1.92	3.43	
SH	1.43 (0.60)	.54	1.88	4.41	.75
(q62)	1.33 (0.65)	.76	2.11	4.40	
(q63)	1.52 (0.70)	.80	1.29	1.37	
SK	1.40 (0.51)	.61	1.77	4.34	.72
(q65)	1.63 (0.75)	.71	1.08	0.74	
(q66)	1.28 (0.55)	.58	2.36	6.94	
(q67)	1.29 (0.58)	.72	2.17	5.07	
ANX	1.66 (0.83)	.58	1.31	1.13	.94
(q69)	1.29 (0.58)	.91	2.17	5.07	
(q70)	1.69 (0.87)	.96	1.16	0.61	
OM (q41)	1.79 (1.00)	.64	1.01	-0.023	--
CO (q46)	1.69 (0.73)	.50	0.89	0.56	--
SC (q59)	1.49 (0.81)	.74	1.61	1.78	--
SN (q64)	1.56 (0.77)	.52	1.27	0.92	--
WL (q68)	1.46 (0.75)	.50	1.77	2.82	--
WO (q71)	1.41 (0.74)	.55	1.80	2.51	--
NEP (q72)	1.60 (0.78)	.45	1.14	0.59	--

*HN-43*  revised European Organization for Research and Treatment of Cancer Quality of Life Questionnaire-Head and Neck Module, *PA* pain in the mouth, *SW* problems with swallowing, *TE* problems with teeth, *DR*  dry mouth and sticky saliva, *SE* problems with senses, *SP* speech problems, *BI* body image problems, *SO* social eating, *SX* sexual problems, *SH* problems with shoulder, *SK* skin problems, *ANX* fear of disease progression, *OM* problems opening mouth, *CO* coughing, *SC* sproblems with ocial contact, *SN* swelling in the neck, *WL* weight loss, *WO* problems with wound healing, *NEP* neurological problems

^a^ Factor loading derived from confirmatory factor analysis Model 1 (please see Table 3 for details)

The CFA indicated that most proposed models had acceptable fit indices, including nonsignificant χ^2^ tests (*p*-values=.19 to .95), except for the SRMR (0.092) in Model 3 (i.e., the model incorporating QLQ-HN43 with EQ-5D). Moreover, the QLQ-HN43 item factor loadings were from moderate to strong (> .45) (Table 2). In addition, the latent factor scores in the QLQ-HN43 were all significantly associated (*r*=.183 to .787; all *p*-values<.01; Supplementary Table S1). The concurrent validity of the QLQ-HN43 subscales was fully supported by the significant and mostly moderate correlations with WHOQOL-BREF (*r*=-.111 to − .508; all *p*-values<.05) as well as with EQ-5D utility in CFA findings (*r*=.276 to .655; all *p*-values<.01). Moreover, the QLQ-HN43 subscales showed relatively higher correlations with more relevant domains in the WHOQOL-BREF. For example, the ‘pain in the mouth’ subscale was associated with the WHOQOL-BREF physical domain (*r*=-.485), and the ‘fear of disease progression’ subscale was associated with the WHOQOL-BREF psychological domain (*r*=-.411) (Table 4).

**Table 3:** Goodness of fit indices in confirmatory factor analysis (CFA) across different quality of life (QoL) models

Model	χ^2^ (df)	*p*-value	CFI	TLI	RMSEA	SRMR
1: HN43	833.437 (848)	.63	1.000	1.001	0.000	0.075
2: HN43+WHOQOL-BREF	347.053 (840)	1.00	1.000	1.032	0.000	0.042
3: HN43+EQ-5D	481.802 (897)	1.00	1.000	1.029	0.000	0.058
4: PA	0.095 (2)	.95	1.000	1.029	0.000	0.010
5: SW	0.322 (2)	.85	1.000	1.016	0.000	0.016
6: SP	0.590 (5)	.99	1.000	1.014	0.000	0.018
7: SO	1.076 (2)	.58	1.000	1.006	0.000	0.029

Only scales of PA, SW, SP, and SO were examined because other scales in the HN43 do not have sufficient item numbers for CFA

*HN-43* revised European Organization for Research and Treatment of Cancer Quality of Life Questionnaire-Head and Neck Module, *WHOQOL-BREF* the World Health Organization Quality of Life Questionnaire Brief version, *EQ-5D* EuroQol Five-Dimension, *PA* pain in the mouth, *SW* problems with swallowing, *SP* speech problems, *BI* body image problems, *SO* social eating problems, *SX* sexual problems, *G* generic factor, *S* specific factor

Model HN43 contained only one specific QoL factor (assessed using HN43)

Model HN43+WHOQOL-BREF contained one specific QoL factor (assessed using HN43) and one generic QoL factor (assessed using WHOQOL-BREF)

Model HN43+EQ-5D contained one specific QoL factor (assessed using HN43) and one generic QoL factor (assessed using EQ-5D)

**Table 4:** Concurrent validity of HN43 with WHOQOL-BREF and EQ-5D derived from confirmatory factor analysis findings

	WHOQOL-BREF						EQ-5D
	Q1^a^	Q2^b^	Phy	Psy	Soc	Env	Utility
PA	-.404**	-.425**	-.485**	-.477**	-.368**	-.425**	.569**
SW	-.388**	-.418**	-.416**	-.466**	-.311**	-.416**	.490**
TE	-.331**	-.285**	-.376**	-.421**	-.328**	-.378**	.412**
DR	-.420**	-.405**	-.384**	-.448**	-.236**	-.344**	.500**
SE	-.195**	-.167**	-.252**	-.244**	-.174**	-.260**	.412**
SP	-.306**	-.223**	-.348**	-.384**	-.315**	-.411**	.498**
BI	-.329**	-.336**	-.381**	-.435**	-.333**	-.447**	.451**
SO	-.508**	-.442**	-.471**	-.496**	-.383**	-.448**	.655**
SX	-.281**	-.271**	-.296**	-.357**	-.314**	-.347**	.278**
SH	-.287**	-.206**	-.412**	-.314**	-.298**	-.405**	.435**
SK	-.344**	-.308**	-.425**	-.414**	-.294**	-.442**	.544**
ANX	-.378**	-.461**	-.366**	-.411**	-.220**	-.285**	.557**
OM	-.246**	-.232**	-.298**	-.349**	-.328**	-.370**	.329**
CO	-.242**	-.247**	-.312**	-.258**	-.111	-.196**	.342**
SC	-.321**	-.313**	-.392**	-.396**	-.321**	-.411**	.471**
SN	-.314**	-.275**	-.403**	-.324**	-.218**	-.333**	.379**
WL	-.293**	-.367**	-.303**	-.270**	-.137*	-.250**	.442**
WO	-.276**	-.308**	-.322**	-.319**	-.264**	-.364**	.539**
NEP	-.280**	-.254**	-.281**	-.263**	-.252**	-.337**	.434**
EQ-5D	-.661**	-.673**	-.816**	-.722**	-.529**	-.591**	--

Values with gray shadows indicate relevant domains’ correlations

*HN-43* revised European Organization for Research and Treatment of Cancer Quality of Life Questionnaire-Head and Neck Module, *WHOQOL-BREF* the World Health Organization Quality of Life Questionnaire Brief version, *EQ-5D* EuroQol Five-Dimension, *Phy* physical domain, *Psy* psychological domain, *Soc* social domain, *Env* environment domain, *PA* pain in the mouth, *SW* problems with swallowing, *TE* problems with teeth, *DR* dry mouth and sticky saliva, *SE* problems with senses, *SP* speech problems, *BI* body image problems, *SO *social eating problems, *SX *sexual problems, *SH* problems with shoulder, *SK* skin problems, *ANX* fear of disease progression, *OM* problems opening mouth, *CO* coughing, *SC* social contact problems, *SN* swelling in the neck, *WL* weight loss, *WO* problems with wound healing, *NEP* neurological problems

^a^ The first item in the WHOQOL-BREF (overall quality of life)

^b^ The second item in the WHOQOL-BREF (general health)

*p<.05; **p<.01

Known-group validity of the QLQ-HN43 was supported by significant differences in QLQ-HN43 scores between participants with stages 0 to III cancer and those with stage IV cancer (Table 5). More specifically, participants with stage IV cancer reported significantly worse QoL than did those with stages 0 to III cancer with a small to moderate effect (*d*=-0.32). Regarding each QLQ-HN43 subscale, significant differences (of which participants with stage IV cancer had worse QoL than did those with stages 0 to III cancer) were found in problems with teeth (*d*=-0.44), dry mouth and sticky saliva (*d*=-0.33), speech problems (*d*=-0.41), swelling in the neck (*d*=-0.43), and neurological problems (*d*=-0.35). Moreover, there were no significant differences in all WHOQOL-BREF domain scores and EQ-5D utility scores between participants with stages 0 to III cancer and those with stage IV cancer (*p*=.06 to .88).

**Table 5:** Known-group validity of HN43 compared with WHOQOL-BREF and EQ-5D by clinical stage

	Mean (SD)		t (*p*-value)/ df	Cohen’s d
	Stages 0 to III(*n* = 111)	Stages IV(*n* = 70)		
HN43	17.17 (16.16)	22.17 (15.29)	2.07 (.04)/ 179	-0.32
TE	27.33 (27.62)	40.32 (33.48)	2.72 (.008)/ 179	-0.44
SN	14.41 (22.75)	25.24 (29.18)	2.64 (.01)/ 179	-0.43
SP	17.90 (24.77)	29.14 (31.58)	2.53 (.01)/ 179	-0.41
NEP	16.52 (23.73)	25.24 (28.05)	2.16 (.03)/ 179	-0.35
DR	29.88 (26.12)	38.57 (27.74)	2.13 (.04)/ 179	-0.33
SX	10.36 (18.81)	16.43 (24.49)	1.77 (.08)/ 179	-0.29
SE	15.02 (24.10)	22.14 (27.47)	1.84 (.07)/ 179	-0.28
SW	20.27 (23.29)	26.90 (27.92)	1.66 (.10)/ 179	-0.27
SO	18.47 (26.62)	25.24 (28.94)	1.61 (.11)/ 179	-0.25
CO	20.12 (22.60)	24.76 (25.81)	1.27 (.21)/ 179	-0.20
OM	24.32 (32.71)	30.00 (33.65)	1.12 (.26)/ 179	-0.17
BI	11.01 (19.04)	13.81 (20.24)	0.94 (.35)/ 179	-0.14
SC	14.11 (24.84)	16.67 (28.23)	0.64 (.52)/ 179	-0.10
SK	12.01 (15.94)	12.86 (14.36)	0.36 (.72)/ 179	-0.06
WL	14.11 (24.01)	15.24 (21.75)	0.32 (.75)/ 179	-0.05
WO	12.31 (24.17)	12.86 (25.56)	0.14 (.89)/ 179	-0.02
SH	14.11 (19.10)	14.29 (22.93)	0.05 (.96)/ 179	-0.01
PA	13.44 (18.91)	13.21 (15.57)	0.08 (.93)/ 179	0.01
ANX	20.57 (25.91)	18.33 (24.92)	0.57 (.57)/ 179	0.09
W_Phy	14.75 (6.19)	14.62 (6.40)	0.37 (.72)/ 173	0.06
W_Psy	13.84 (6.47)	13.13 (6.34)	1.88 (.06)/ 173	0.29
W_Soc	14.15 (6.12)	14.10 (5.91)	0.15 (.88)/ 173	0.02
W_Env	14.23 (6.06)	14.11 (6.06)	0.40 (.69)/ 173	0.06
EQ-5D	0.94 (0.12)	0.96 (0.08)	1.78 (.08)/ 175	0.26

Higher QLQ HN43 total and scale scores indicate worse quality of life

*HN-43 * revised European Organization for Research and Treatment of Cancer Quality of Life Questionnaire-Head and Neck Module, *WHOQOL-BREF*   the World Health Organization Quality of Life Questionnaire Brief version, *EQ-5D* EuroQol Five-Dimension, *df* degree of freedom, *PA* pain in the mouth, *SW* problems with swallowing, *TE* problems with teeth, *DR*   dry mouth and sticky saliva, *SE* problems with senses, *SP* speech problems, *BI* body image problems, *SO *social eating problems, *SX* sexual problems, *SH* problems with shoulder, *SK* skin problems, *ANX* fear of disease progression, *OM* problems opening mouth, *CO* coughing, *SC* social contact, *SN* swelling in the neck, *WL* weight loss, *WO* problems with wound healing, *NEP* neurological problems, *W_Phy* WHOQOL-BREF physical domain, *W_Psy* WHOQOL-BREF psychological domain, *W_Soc* WHOQOL-BREF social domain, *W_Env* WHOQOL-BREF environment domain

## Discussion

The present study assessed the psychometric properties of the QLQ-HN43 as a specific instrument to examine QoL among individuals with HNCs. Promising results were found regarding the validity and reliability of the QLQ-HN43 among Taiwanese individuals. Moreover, incorporating two generic QoL instruments (i.e., WHOQOL-BREF and EQ-5D) with the QLQ-HN43 showed a good fit in the CFAs. However, although WHOQOL-BREF and EQ-5D may provide valuable information on the state of QoL among individuals with HNCs, the EQ-5D may not be sensitive enough to detect QoL difficulties among individuals with HCNs because of the poor associations between the EQ-5D and QLQ-HN43. However, the WHOQOL-BREF is recognized as a commonly used generic QoL instrument that can be used together with the specific QoL instrument (e.g., the QLQ-HN43).

As reported in the original QLQ-HN43 development study, the scale showed acceptable structure and internal consistency in accordance with the present study’s findings. The developers reported Cronbach’s α for subscales ranging from .64 to .89, comparable to those here (α = .68 to .94) (Singer et al., 2019). Similarly, the internal consistency was moderate to high in the Serbian and Marathi versions of the scale (Trivic et al., 2016; Waghmare et al., 2020). CFAs in the present study assessed different models and found appropriate fit indices for the QLQ-HN43, as well as when the QLQ-HN43 was incorporated with generic QoL instruments (i.e., WHOQOL-BREF and EQ-5D). As aforementioned, almost all CFA fit indices indicated good fit indices, whereas, in the original study, the authors reported moderate values for such indices (Singer et al., 2019). However, similar to the original study and other scale validations, the indices in the present study indicated the scale had an acceptable factor structure as hypothesized by the original scale developers.

Despite previous validations of the scale that simply used a recommended accompanying general cancer QoL scale (i.e., QLQ-C30) for assessing other psychometric properties of the scale (Trivic et al., 2016; Waghmare et al., 2020), the present study innovatively used generic QoL instruments (i.e., WHOQOL-BREF and EQ-5D) and found interesting results. First, it was found that incorporating the WHOQOL-BREF with QLQ-HN43, as shown in the CFA model, may provide an acceptable structure with good fit indices. Second, it was found that a short and brief scale (such as the EQ-5D), may also be incorporated with the QLQ-HN43. At the same time, the EQ-5D may be suggested as the second most appropriate scale after WHOQOL-BREF due to slightly lower acceptable values in fit indices. These are new findings in validating the QLQ-HN43 that previous studies did not report.

In the original QLQ-HN43 development study, as well as the Serbian and Marathi validation studies, a recommended general cancer QoL scale (i.e., QLQ-C30) was used to assess concurrent validity. Singer et al. (2019) found that the size and directions of the correlations between all subscales of these measures were in accordance with the conceptual model, and they did not find any correlations greater than .7, showing these scales were independent of each other and could not be replaced interchangeably. The present study’s findings, in line with prior validations of the QLQ-HN43, confirmed its concurrent validity. However, the present study examined these criteria through other QoL instruments (i.e., WHOQOL-BREF and EQ-5D), and the findings also corroborated previous findings regarding the robust concurrent validity of the QLQ-HN43 (Trivic et al., 2016; Waghmare et al., 2020).

Another aspect related to the known-group validity of the scale that was also examined in the previous studies was the capability of the scale to be used among individuals with different conditions or characteristics, such as those with severe forms of HNCs vs. those with less severe forms. As found in the present study, the QLQ-HN43 differentiated between participants at stages I to III of the disease and those at stage IV, indicating lower QoL than those at earlier stages of cancer. However, some conditions, such as problems with teeth, dry mouth, sticky saliva, speech, swelling in the neck, and neurological problems, had significant roles in discriminating between those with and without these problem conditions. This may show that these problems may have important effects on worsening the QoL and should receive special attention.

Singer et al. (2019) also reported results consistent with the present study’s findings. They reported that problems such as swelling in the neck, skin problems, and dry mouth may be used as criteria to differentiate between subgroups of the participants (i.e., those with Stage I-III cancer vs. those with Stage IV cancer). However, the present study found that several subscales in QLQ-HN43, such as pain in the mouth, body image problems, problems with the shoulder, and problems with wound healing, did not show any significant differences between those in earlier stages of the disease versus those in the most advanced stage. The likely explanation for this finding may be related to the amount of influence that each symptom or problem may have on the participant’s QoL. In other words, some problems may be greater than others in affecting QoL, and as aforementioned, these problems should be attended to by healthcare providers as the more important variables to provide care, and to conduct interventions to improve QoL among these individuals. Moreover, WHOQOL-BREF domain scores and the EQ-5D utility score showed no significant differences between the participants with different cancer stages. In this regard, the QLQ-HN43 in clinical or research settings could be more sensitive and outperform WHOQOL-BREF and EQ-5D in assessing patients’ QoL.

Another notable finding of the present study that was relatively unexpected, was a non-significant association between the EQ-5D scores and QLQ-HN43 scores (whereas almost all subscale scores of QLQ-HN43 were significantly correlated with WHOQOL-BREF scores). Indeed, only a few QLQ-HN43 subscales, such as speech problems and problems with opening the mouth, were correlated to the utility score of EQ-5D, and only problems with wound healing indicated a significant association with the visual analogue scale of the EQ-5D. There are two likely explanations for such findings. First, the QLQ-HN43 assesses various symptoms that may not have a significant impact on general QoL, and these scores may not appropriately match specified subscales assessed by the EQ-5D, such as mobility, self-care, usual activities, pain, and depression.

Second, the lack of associations between these two measures may relate to ceiling effects. More specifically, the EQ-5D was developed with only five items, with each item rating on a three-level scale based on utility theory. Prior evidence shows that the EQ-5D is more likely to have ceiling effects as compared to QoL instruments developed based on psychometric theory (e.g., WHOQOL-BREF) (Feng et al., 2021; Kangwanrattanakul & Parmontree, 2020). Indeed, participants in the present study had an EQ-5D utility score of 0.944 (out of a 0–1 scale), whereas their WHOQOL-BREF domain scores were between 58.02 and 65.08 (out of a 0-100 scale). When almost all participants had the highest utility scores in the EQ-5D, the narrow score distribution lowered the overall correlation coefficient with another QoL score (i.e., the QLQ-HN43 in the present study).

### Implications, strengths, and limitations

Some implications can be proposed using the present study’s findings. First, the QLQ-HN43 can be incorporated or used alongside the WHOQOL-BREF to obtain a detailed profile of the QoL for individuals with HNCs. Second, the EQ-5D may not be a good instrument for assessing QoL for individuals with HNCs. Third, the QLQ-HN43 itself can efficiently assess different levels of cancer-specific domains of QoL.

Aside from the strengths of the study, such as being the first study to incorporate the WHOQOL-BREF with the QLQ-HN43, and the use of larger sample sizes than previous cultural validation studies of QLQ-HN43, some limitations of the present study should be addressed. First, participants were recruited from only one medical center in Taiwan and not all of these wanted to participate in the study (which used convenience sampling to recruit volunteer participants). Therefore, the findings may not generalize to all Taiwanese individuals with HNCs.

Second, a cross-sectional design was used and was unable to assess the responsiveness of the QLQ-HN43 to changes, as examined in the original study. Therefore, further investigation is needed to show how robust the QLQ-HN43 is in detecting changes in participants’ QoL across the time of treatment or survival. Third, the general cancer QoL instrument (i.e., QLQ-C30) was not used to examine the concurrent validity of the QLQ-HN43. Although the present study used WHOQOL-BREF and EQ-5D, evaluating the QLQ-C30 alongside the QLQ-HN43 may have also provided additional comparative information regarding these general scales that can be used among individuals with HNCs. However, to prevent data collection being a time-consuming process for the participants, the QLQ-C30 was not used because prior studies have already used it, showing evidence of its good psychometric properties.

Finally, to assess the known-group validity of the scale, individuals were categorized into two categories based on the severity of cancer problems (i.e., mild to moderate vs. severe). However, the present study did not have information on other criteria to examine the known-group validity (e.g., type of treatment; survivors vs. those still undergoing treatment). Future studies should examine how much the QLQ-HN43 differentiates individuals with HNCs in relation to other clinical characteristics.

## Conclusion

The present study showed that the QLQ-HN43 is a valid and reliable instrument for assessing QoL among Taiwanese individuals with HNCs. Moreover, the feasibility of using other generic QoL instruments (e.g., WHOQOL-BREF and EQ-5D) to gather supplemental information on different aspects of QoL was verified. However, because the EQ-5D did not show any significant correlations with many of the QLQ-HN43 subscales, applying it as a brief but effective measure to collect information on general QoL among these individuals needs further investigation and should be used with caution. However, using WHOQOL-BREF alongside the QLQ-HN43 may be helpful for healthcare providers to obtain comprehensive QoL information. Also, it is recommended that healthcare providers use these general and specific scales to monitor their clients in terms of QoL and plan practical interventions to address the most affected components of QoL among these individuals.

## Supplementary Information

Supplementary Material 1: Supplementary Table S1. Factor associations of HN43 in confirmatory factor analysis (N=290).

## Data Availability

The datasets used and/or analyzed during the current study are available from the corresponding author on reasonable request.

## Abbreviations

EORTC: European Organization for Research and Treatment of Cancer
QLQ-HN43: Quality of Life Questionnaire-Head and Neck Module 43-item version
CFA: Confirmatory factor analysis
WHOQOL-BREF: World Health Organization Quality of Life Questionnaire Brief version
EQ-5D: EuroQol Five-Dimension
HNC: Head and neck cancer
QoL: Quality of life
WHO: World Health Organization
QLQ-C30: General Quality of Life Questionnaire Core 30
CFI: Comparative fit index
TLI: Tucker-Lewis index
RMSEA: Root mean square error of approximation
SRMR: Standardized root mean square residual
