# Supplementary material for: Validating cancer-specific and generic quality of life (QoL) instruments among patients with head and neck cancers: incorporating the European Organization for Research and Treatment of Cancer Quality of Life Questionnaire-Head and Neck Module with generic QoL instruments
Source: Psicol Reflex Crit. 2026 May 6;39:20. doi: 10.1186/s41155-026-00394-1 (PMC13272734; doi:10.1186/s41155-026-00394-1)
Supplement: Supplementary file 1 — Supplementary Material 1: Supplementary Table S1. Factor associations of HN43 in confirmatory factor analysis (N=290). [file 41155_2026_394_MOESM1_ESM.docx]

Supplementary Table S1. Factor associations of HN43 in confirmatory factor analysis (N=290)

|  | PA | SW | TE | DR | SE | SP | BI | SO | SX | SH | SK | ANX | OM | CO | SC | SN | WL | WO | NEP |
| --- | --- | --- | --- | --- | --- | --- | --- | --- | --- | --- | --- | --- | --- | --- | --- | --- | --- | --- | --- |
| PA | -- |  |  |  |  |  |  |  |  |  |  |  |  |  |  |  |  |  |  |
| SW | .554 | -- |  |  |  |  |  |  |  |  |  |  |  |  |  |  |  |  |  |
| TE | .398 | .677 | -- |  |  |  |  |  |  |  |  |  |  |  |  |  |  |  |  |
| DR | .548 | .705 | .538 | -- |  |  |  |  |  |  |  |  |  |  |  |  |  |  |  |
| SE | .329 | .564 | .344 | .569 | -- |  |  |  |  |  |  |  |  |  |  |  |  |  |  |
| SP | .327 | .666 | .611 | .434 | .469 | -- |  |  |  |  |  |  |  |  |  |  |  |  |  |
| BI | .395 | .543 | .499 | .454 | .272 | .466 | -- |  |  |  |  |  |  |  |  |  |  |  |  |
| SO | .497 | .787 | .708 | .658 | .467 | .677 | .538 | -- |  |  |  |  |  |  |  |  |  |  |  |
| SX | .253 | .361 | .290 | .268 | .276 | .410 | .473 | .374 | -- |  |  |  |  |  |  |  |  |  |  |
| SH | .312 | .342 | .301 | .326 | .302 | .450 | .409 | .369 | .342 | -- |  |  |  |  |  |  |  |  |  |
| SK | .500 | .454 | .329 | .489 | .310 | .353 | .591 | .439 | .357 | .424 | -- |  |  |  |  |  |  |  |  |
| ANX | .443 | .432 | .339 | .400 | .369 | .369 | .508 | .468 | .235 | .440 | .494 | -- |  |  |  |  |  |  |  |
| OM | .497 | .557 | .583 | .530 | .257 | .457 | .487 | .498 | .228 | .386 | .366 | .323 | -- |  |  |  |  |  |  |
| CO | .350 | .514 | .296 | .457 | .474 | .423 | .269 | .363 | .197 | .269 | .360 | .293 | .193\| | -- |  |  |  |  |  |
| SC | .285 | .587 | .574 | .335 | .363 | .735 | .586 | .661 | .476 | .506 | .395 | .412 | .418 | .300 | -- |  |  |  |  |
| SN | .432 | .365 | .298 | .448 | .311 | .317 | .351 | .382 | .272 | .620 | .489 | .413 | .340 | .259 | .312 | -- |  |  |  |
| WL | .275 | .354 | .261 | .404 | .343 | .310 | .387 | .446 | .223 | .401 | .491 | .598 | .246 | .232 | .389 | .422 | -- |  |  |
| WO | .469 | .376 | .339 | .307 | .365 | .343 | .487 | .378 | .194 | .501 | .540 | .645 | .330 | .326 | .422 | .412 | .427 | -- |  |
| NEP | .414 | .277 | .311 | .329 | .183 | .294 | .386 | .279 | .273 | .439 | .527 | .331 | .263 | .205 | .276 | .373 | .289 | .408 | -- |

Note. all *p*-values<.01. HN-43=revised European Organization for Research and Treatment of Cancer Quality of Life Questionnaire-Head and Neck Module; PA=pain in the mouth; SW=problems with swallowing; TE=problems with teeth; DR=dry mouth and sticky saliva; SE=problems with senses; SP=speech problems; BI=body image problems; SO=social eating problems; SX=sexual problems; SH=problems with shoulder; SK=skin problems; ANX=fear of disease progression; OM=problems opening mouth; CO=coughing; SC=social contact; SN=swelling in the neck; WL=weight loss; WO=problems with wound healing; NEP=neurological problems.
